# Can Acupuncture Improve the Flexibility of Hamstring Muscles? A Randomized, Blinded, and Controlled Pilot Study

**DOI:** 10.3390/healthcare11040490

**Published:** 2023-02-08

**Authors:** Rui Miguel Carvalho, Jorge Machado, Maria João Santos, Luís Carlos Matos

**Affiliations:** 1 ICBAS—Institute of Biomedical Sciences Abel Salazar, University of Porto, 4050-313 Porto, Portugal; 2 CBSin—Center of BioSciences in Integrative Health, 4405-604 Vila Nova de Gaia, Portugal; 3 LABIOMEP—Porto Biomechanics Laboratory, University of Porto, 4200-450 Porto, Portugal; 4 Escola Superior de Saúde Jean Piaget, 4405-678 Vila Nova de Gaia, Portugal; 5 Faculdade de Engenharia da Universidade do Porto, 4200-465 Porto, Portugal; 6 CTEC—Centro Transdisciplinar de Estudos da Consciência, Universidade Fernando Pessoa, 4249-004 Porto, Portugal

**Keywords:** muscle flexibility, acupuncture, traditional Chinese medicine, Heidelberg model of TCM, placebo

## Abstract

(1) Background: The lack of flexibility is frequently reported as a risk factor for hamstring muscle damage. Acupuncture, a therapeutic tool of traditional Chinese medicine (TCM), may play a role in both treatment and prevention by improving muscle strength, microcirculation, and reducing muscle soreness. The primary objective of this pilot study was to examine the immediate effects of acupuncture on hamstring muscle stretching and on the pain or discomfort reported during stretching. (2) Methods: To mitigate heterogeneity effects, and due to the small sample size, the study employed a crossover design in which each participant was tested at three different moments of the experimental period with verum (true acupuncture in selected acupoints), sham (fake acupuncture in zones of the skin not corresponding to any known acupoint but near the selected acupoints), and placebo (stimulation of the selected acupoints with a stainless steel wire and cannula, without puncturing) stimulations. Flexibility and pain or discomfort were assessed using the seat and reach test (SR) and a visual analogic scale (VAS). (3) Results: Significant changes in flexibility were observed after verum acupuncture (*p* = 0.03), while no significant changes were seen in sham and placebo (*p* = 0.86 and *p* = 0.18, respectively). No significant differences were found in pain or discomfort during any of the stimulations (verum, *p* = 0.55; sham, *p* = 0.50; placebo, *p* = 0.58). (4) Conclusions: The results of this pilot study suggest that acupuncture may improve flexibility in the hamstring muscles, though it does not significantly affect pain or discomfort during stretching.

## 1. Introduction

The tendency of hamstring muscles to shorten is well-known. This is due to their multi-joint character, tonic postural nature, and the tensional forces to which they are constantly exposed [1,2]. The literature indicates that a lack of hamstring flexibility can result in significant muscle imbalances, predisposing athletes to muscle injuries [3], patellar tendinopathy, and patellofemoral pain [4,5,6], as well as potentiating pain in the lumbar region [2,5,6]. Thus, the flexibility of the hamstrings is of vital importance in preventing injuries and improving quality of life.

Flexibility, traditionally understood as a joint range of motion, has been considered a critical aspect of physical fitness and health since the 1980s. The benefits of flexibility, which can be gained from stretching exercises, include improved range of motion and function, enhanced athletic performance, decreased risk of injury, prevention or reduction of post-exercise soreness, and improved coordination. The literature has established a link between poor hamstring flexibility and low back pain in adolescents, and a higher lifetime risk of low back pain has been associated with this condition [7].

The hamstring muscle group (consisting of the biceps femoris, semitendinosus, and semimembranosus) is particularly susceptible to injury in sports that require high-velocity movements, such as rugby, basketball, American football, track and field, and soccer. Hamstring muscle injury is a significant concern in professional football, as it is the most common non-contact injury, accounting for 12% to 16% of all injuries. Furthermore, the recurrence rate is relatively high, with a reported incidence of 13%. Additionally, more than half of the injuries result in an interruption of 8 to 28 days for recovery. [8].

Age and prior muscle injuries are well-established non-modifiable risk factors for hamstring muscle injuries. In contrast, modifiable risk factors include deficits in eccentric hamstring strength, hamstring–quadriceps strength, biceps femoris fasciculus length, and hamstring flexibility. Low flexibility is one of the most significant risk factors described in the literature for primary and recurrent hamstring muscle injuries. As an illustration, in preseason testing, soccer teams often conduct assessments of hamstring flexibility to identify players who may be more susceptible to injury and to prevent potential hamstring injuries [7,8].

Due to its benefits, stretching is often used in physical activity and the rehabilitation of patients with musculoskeletal problems. The literature suggests that programs aimed at increasing flexibility can reduce the severity and frequency of injuries, enhance performance in sports and overall physical fitness, expand the range of motion of joints, increase tissue elasticity, and alleviate pain [9,10].

Acupuncture, a therapeutic modality of traditional Chinese medicine (TCM), has physiological and psychological effects, which are characterized as either specific or non-specific. The specific effects refer to the analgesic effects achieved by puncturing a particular location to an appropriate depth for a suitable duration and number of treatment sessions [11]. Non-specific psychological effects are related to patients’ perceptions, beliefs, experiences, and expectations [12]. Hence, simulated acupuncture is required to assess the specific effects of acupuncture. Sham and placebo refer to any control procedure employed in blind treatment evaluations in acupuncture clinical trials. There are various sham procedures available, including the puncturing of non-acupuncture points, superficial skin penetration at acupuncture points, and non-penetration of acupuncture points with sham needle devices [13,14].

Acupuncture has been shown to have positive effects on muscle strength [15] and microcirculation [16]. It also exhibits anti-inflammatory properties [17] and can inhibit spinal and supraspinal nociceptive transmission [18]. Therefore, acupuncture may represent an effective, rapid, and valuable treatment for muscle soreness after physical exercise, enhancing the performance of athletes and the productivity of workers and reducing the duration of the rehabilitation process [19].

The literature presents conflicting data regarding the effectiveness of acupuncture on post-exercise pain. Itoh et al. (2010) found significant differences in visual analog scores for pain between a control group and a tender point group immediately after acupuncture and three days after exercise [20]. Meanwhile, Lin and Yang (1999) reported that muscle soreness perception was significantly lower 72 h after treatment in the acupuncture-group compared to the control group [21]. However, Barlas et al. (2000) found weak evidence of acupuncture’s effect on pain, concluding that it has little effect on the cardinal signs and symptoms of delayed onset muscle soreness (DOMS) under their experimental conditions, after comparing real acupuncture in classic and tender point, sham acupuncture, and no-treatment groups [22].

The Heidelberg model of TCM is an integrated neurovegetative model that is complementary and interactive with Western medicine. This model translates the structural concepts of TCM language into Western physiology, thus enabling the rational use of reflex therapeutic systems, anti-inflammatory mechanisms, and mental training, which are applied in techniques such as acupuncture and “Qigong” [23]. According to the Heidelberg model, the lack of flexibility of the hamstrings is related to Stage I in Algor Leadens Theory (ALT) (Yang major in Shang Han Lun), with pain upon stretching along the vesical (bladder) conduit being one of the signs. At this stage, the agent algor (cold) affects the “wei qi” found in the extima (exterior), resulting in a decrease in “xue” (a concept similar to blood) in the local area, which reflects a decrease in the microcirculation [24,25,26,27].

Despite the existence of literature on the effect of acupuncture on muscle strength and pain, there is limited research regarding its impact on hamstring flexibility. Bearing this in mind, the main goal of this pilot study was to examine the immediate effects of acupuncture on hamstring muscle stretching and the pain or discomfort reported during stretching.

## 2. Materials and Methods

This study adopted a randomized, controlled, crossover experimental design, and was approved by the Ethics Committee of the Institute of Biomedical Sciences Abel Salazar (reference 2020/CE/P020(P332/CETI/ICBAS)). A convenience sample of 15 healthy volunteers, comprised of nine males, and six females, aged between 18 and 35, was used. The volunteers were informed about the study procedures and voluntarily participated after completing a short questionnaire and signing an informed consent form.

All volunteers were instructed to abstain from physical exercise for a period of one week before and during the study.

Inclusion criteria: healthy volunteers aged between 18 and 35, capable of following basic instructions, and abstaining from physical exercise for a period of one week before and during the study, were eligible.

Exclusion criteria: subjects with needle phobia, who had engaged in physical exercise within the previous 7 days, with previous hamstring injuries, diagnosed with sciatica or low back pain, with orthopedic prostheses (knee or hip), and reporting any relevant impact accident (road accident, for example), were excluded from this study.

### 2.1. Data Collection Instruments

- Socio-demographic questionnaire: This tool is used to characterize the sample regarding social data, such as gender, age, profession, and current professional situation;
- VAS Questionnaire: The Visual Analogue Scale (VAS) is a measurement instrument used to measure a characteristic or attitude that changes across a continuum of values that cannot be easily quantified. Pain can be assessed using VAS with a numeric linear scale from 0 to 10. On this scale, “zero” is defined as “no pain”, and the extreme value of “ten” is considered “very severe pain or unbearable pain”.

**Figure 1:**
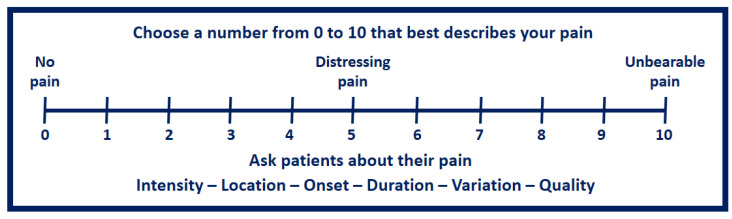
Visual analogue scale (VAS) for assessing pain (adapted from [29]).

The VAS is often represented as a horizontal straight line of approximately 100 millimeters that, along its length, has words, images, or both that define the type of pain [28]. A typical representation of a VAS scale is shown in Figure 1.

**Figure 2:**
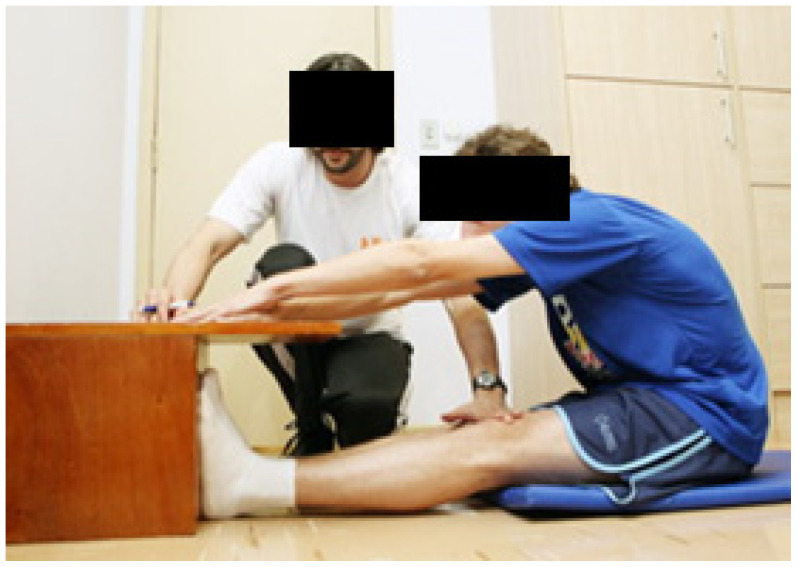
Sit and reach test to assess flexibility.

- Muscle flexibility test: The sit and reach (SR) test, based on the procedure proposed by Ayala et al. (2012), was employed to measure the flexibility of the hamstring muscles. A wooden box measuring 30.5 cm in height, equipped with a centrally located sliding ruler, was utilized for this test [30]. The zero point was established as the 35 cm mark on the ruler, where the subjects’ feet made contact with the box. This apparatus allows a scoring range from 0 cm (indicating very low flexibility) to 50 cm (indicating very high flexibility). The test was performed with the participants sitting on the floor, with their knees extended in a straight line with bare feet against the vertical edge of the SR box. They were instructed to perform forward hip flexion, with overlapping hands, to reach the maximum point on the scale and maintain the position for 2 s. Two attempts were made, and the result was considered the average of the two evaluations (Figure 2).

### 2.2. Procedures

The participants were divided into three groups in a crossover experimental design, receiving the treatments in random order in 3 different periods: (a) a real acupuncture group—experimental group (EG); (b) a fake acupuncture or sham acupuncture group, which served as a true control group (CG); (c) a simulated acupuncture group (cannula and wire) that served as the placebo group (PG). Sham is understood as fictitious control, i.e., invasive procedures, including subcutaneous insertion of superficial needles, in areas without recognized acupoints [31].

**Figure 3:**
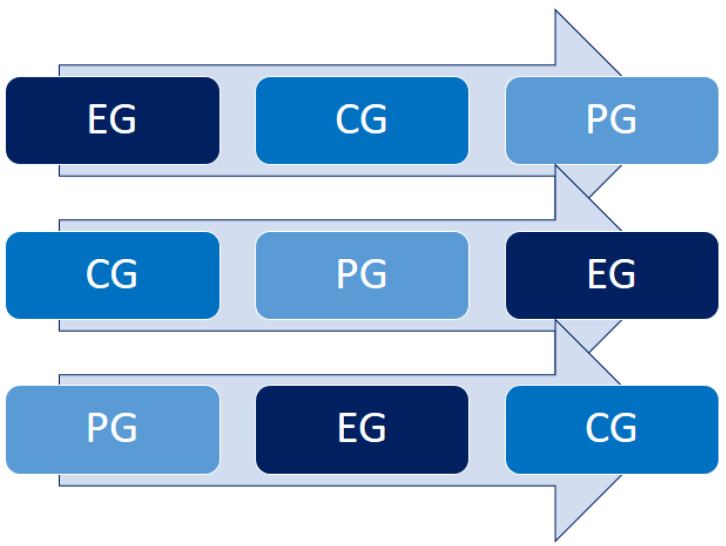
Intervention sequences of the crossover design.

At the beginning of the experimental period, each subject was randomly assigned to each intervention/group by selecting one of 15 papers indicating the sequence, without prior knowledge of the result. The three possible sequences are depicted in Figure 3.

A 7-day washout period was employed between the interventions to eliminate any temporary impacts on the physiological and psychological state of the participants [32]. This duration was selected based on available literature and the participants’ availability during the pandemic crisis.

Two evaluation moments were considered on each day of the intervention: T0, before the intervention, and T1, following the intervention. All participants underwent a quantitative and qualitative assessment before and after treatment. The parameters used to establish a comparison were pain/discomfort during stretching, assessed using VAS, and hamstring flexibility, measured using the SR test.

**Figure 4:**
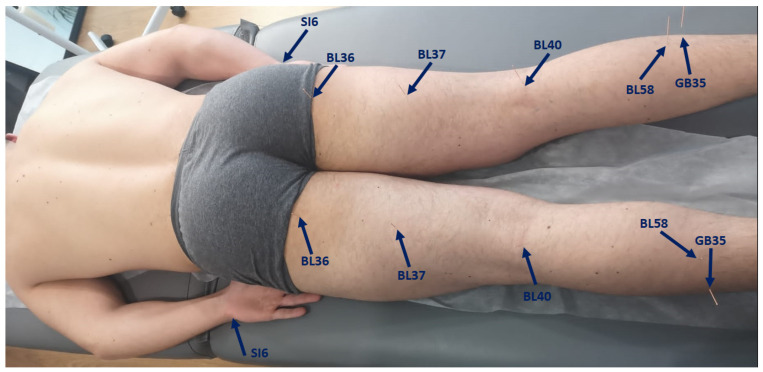
Subject receiving acupuncture treatment (blue arrows point to the inserted needles).

The levels of discomfort/pain were evaluated after the SR test. While undergoing the intervention protocols (true, sham, and placebo interventions), participants were positioned in ventral decubitus on a table with a facial hole, ensuring that they were unable to observe the procedure (Figure 4).

Qualified TCM practitioners selected acupoints based on the Heidelberg model of TCM [33]. The acupoints selected in this study were BL36, BL37, BL40, BL58, SI6, and GB35, bilaterally. The selection of these acupoints was based on the criteria and specificities of each point following the model used in this study. Thus, BL36, or “Chengfu”, opens the bladder channel, relieves pain, and relaxes the tendons; BL37, or “Yinmen”, relaxes the tendons, opens the channel and “luo” vessels, and benefits the lower back; BL40, or “Weizhong”, acts on all affections of the bladder channel, whether due to humor (humidity), algor (cold), or ventus (wind), such as pain in the back, hamstrings, and calves; BL58, or “Feiyang”, opens the channel and “luo” vessels, relieves pain, expels pathogenic factors from “Taiyang” channels (SI, BL), and harmonizes; SI6, or “Yanglao”, relaxes the wood phase and nerves, opens the channel, relieves pain and acute conditions; and GB35, or “Yangjiao”, clears heat, opens the channel, relaxes tendons, regulates gallbladder “qi”, and calms the “shen” (spirit).

The location of the acupuncture points on the body was determined using the proportional unit of measurement “cun” in relation to each individual. False or sham acupuncture points were selected as generic points on the skin near true acupuncture points but not coincident with any known acupuncture point.

In both the experimental and control groups, acupuncture was performed using disposable, single-use sterilized stainless steel needles (0.25 × 0.25 mm, brand Tewa), after cleaning the skin with alcohol. The type of needle manipulation and insertion depth was kept identical during all sessions. Although needle manipulation techniques are based on subtle variations in lifting and thrusting, twisting, and rotating the needle, which is attributed to reinforcement or reducing power as a function of the frequency and displacement, in this work, to ensure practical reproducibility, the needles were inserted without those variations and remained in place for 20 min. In the placebo group, the skin was also disinfected with alcohol, and the true points were superficially stimulated by touching the skin with the cannula and stainless steel wire The wire did not penetrate the skin and did not remain in place during the resting period. After stimulation, the participant was allowed to rest for 20 min.

### 2.3. Statistical Analysis

Because no prior knowledge existed regarding the variables of interest in the population, the Wilcoxon matched-pairs test, a nonparametric method, was used to compare two sets of observations. The *t*-test for dependent samples was also employed because the observations being compared were based on the same subjects tested in sequence (e.g., before and after each intervention), and a significant portion of the within-group variation in each assessment can be attributed to the individual differences between subjects [34]. The results of both nonparametric and parametric tests were evaluated concerning the statistical significance of the differences between groups. Statistical tests were conducted using the Statistica software for Windows version 7.0.

## 3. Results 

**Figure 5:**
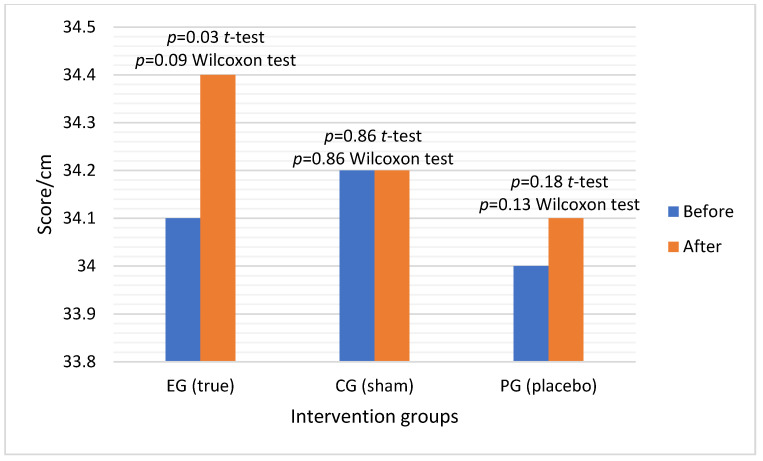
Average flexibility results before and after real, sham, and placebo interventions.

The results of the Student’s *t*-test revealed significant differences in hamstring flexibility measurements (Figure 5) before and after the intervention with true acupuncture (*p* = 0.03). Conversely, in the case of sham acupuncture and placebo, no significant differences were observed, with *p* = 0.86 and *p* = 0.18, respectively. The Wilcoxon test produced results that were consistent in trend, although the statistical significance level for true acupuncture was greater than 0.05, which is commonly accepted as the threshold of significance. The results of the Wilcoxon test were *p* = 0.09, *p* = 0.86, and *p* = 0.13 for true, sham, and placebo acupuncture, respectively.

It is important to note that in the case of sham acupuncture, the observed differences before and after the intervention were the least relevant, as indicated by the high *p*-values in both tests. Although the differences in the placebo group were not considered statistically significant, the *p*-values were lower, suggesting that the intervention may have had some impact on the measured variable. On the other hand, the results indicate that true acupuncture had a significant effect on the measured variable.

**Figure 6:**
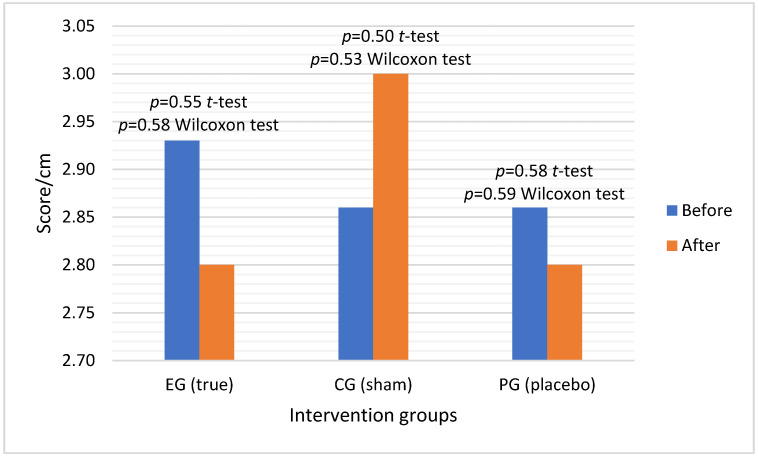
Average VAS results before and after true, sham, and placebo interventions.

Regarding the results of the *t*-test on the VAS scale (Figure 6), the *p*-values were 0.55, 0.50, and 0.58 for true, sham, and placebo acupuncture, respectively. The significance level determined by the Wilcoxon test was also 0.58, 0.53, and 0.59 for true, sham, and placebo acupuncture, respectively. These results show that there were no statistically significant changes in any of the groups.

It is noteworthy to mention that, despite the differences not being statistically significant, there was a decrease in pain/discomfort in both true acupuncture and placebo groups (4.5% and 2.3%, respectively). Conversely, in the sham acupuncture group, there was an increase in pain/discomfort during stretching (4.7%).

## 4. Discussion

The literature reports the positive effects of acupuncture in treating musculoskeletal conditions, however, many studies often lack a thorough analysis of the impact of sham and/or placebo interventions [35,36,37]. To address this limitation, our experimental design included both sham and placebo groups. The differences between groups were evaluated to determine the immediate effects of acupuncture on hamstring flexibility. Our study aimed to determine if the pain intensity during hamstring stretching was affected by acupuncture and if it influenced the flexibility of those muscles.

The sham (CG) and placebo (PG) groups served as control groups. The results in these groups indicate different effects, supporting the hypothesis of a specific physiological response when the stimulation is performed at specific points on the skin, corresponding to acupuncture points. The results show that the stimulation with true acupuncture and using a cannula and wire (placebo) were more effective than skin puncture in areas that do not correspond to any known acupoint (sham), even if the areas were close to the selected acupoints. This suggests that the effects of acupuncture do not solely depend on the stimulation of dermatome zones, but also on the stimulation of unique structures in the skin corresponding to the acupuncture points described in TCM.

Regarding flexibility, the experimental (EG) and placebo (PG) groups produced notable results, however, no significant changes in pain/discomfort were observed during the flexibility test.

The literature shows that a light skin touch can stimulate mechanoreceptors coupled to slow-conducting unmyelinated (C) afferents, leading to activity in the insular region but not in the somatosensory cortex [38,39]. This "limbic touch" response elicited by tactile C results in emotional and hormonal reactions. In many acupuncture studies, control procedures that are intended to be inactive may activate these tactile C afferents, leading to the relief of pain [40]. Our results align with this finding. Indeed, the effects observed in the placebo group (PG) were notable and closer to those in the experimental group (EG) regarding an increase in hamstring flexibility and a decrease in pain/discomfort during stretching, even if these changes were not statistically significant.

The alleviation of muscle pain symptoms may be linked to the stimulation of the neurovegetative system by acupuncture, resulting in an analgesic effect by activating pain-suppressing centers located in the medulla, pons, and midbrain. This stimulation triggers the release of endogenous opioid substances, such as enkephalin and endorphin, into the bloodstream. The endogenous opioids modulate pain perception through their actions on opioid receptors, resulting in analgesic effects and influencing the perception of nociceptive stimuli [41]. Despite several studies reporting the positive effects of acupuncture in reducing muscle pain [42,43], our results showed no significant change in pain/discomfort during hamstring stretching. However, our results were still relevant, as both the experimental group (EG) and placebo group (PG) showed a decrease in this variable, while the sham group (CG) showed an increase.

### Study Limitations

This study has some limitations that should be considered when evaluating its results, such as its experimental nature and time constraints.

The Leopard spot technique is considered the best puncture technique for achieving immediate results [44], but due to the limited sample size and to avoid losing subjects, regular acupuncture with a 20-min rest period was chosen. This approach was less intense than the Leopard spot technique as it only punctured a few points on both sides, which may explain the lack of significant results for pain/discomfort. It would be beneficial for future studies to explore alternative acupuncture methods.

Due to the COVID-19 pandemic, it was difficult to recruit a larger sample. This was due to the need for individualized interventions, which took many days to collect data, and also due to the fear of contamination.

This study evaluated the immediate effects of acupuncture, but future studies would benefit from assessing the long-term impact of acupuncture on both flexibility and pain/discomfort during hamstring stretching.

## 5. Conclusions

Despite the limitations of this study, our results indicate that acupuncture may be a promising approach for enhancing hamstring flexibility. Indeed, we observed improvements in flexibility, although there were no significant changes in pain/discomfort during stretching. According to our results and previous research, acupuncture could aid in preventing hamstring muscle injuries by increasing flexibility, reducing low back pain, and decreasing the likelihood of sports injuries. Therefore, acupuncture can be a valuable technique in the treatment of hamstrings to prevent injuries and improve low back pain, thus reducing the prevalence of these health limitations in society.

While this study was conducted with a small convenience sample of fifteen volunteers, the results are still meaningful concerning the improvement in flexibility, for which the followed methodology, based on the Heidelberg Model of TCM, had an immediate and relevant impact.

## Data Availability

Not applicable.
